# Reduced Left Atrial Appendage Flow Velocity as a Risk of Thromboembolic Events After Catheter Ablation of Atrial Fibrillation

**DOI:** 10.1002/joa3.70233

**Published:** 2025-12-10

**Authors:** Shintaro Yamagami, Satoshi Shizuta, Munekazu Tanaka, Shushi Nishiwaki, Takanori Aizawa, Akihiro Komasa, Takashi Yoshizawa, Tetsuma Kawaji, Chihiro Ota, Naoaki Onishi, Yasuhiro Sasaki, Mitsuhiko Yahata, Kentaro Nakai, Mamoru Hayano, Tetsushi Nakao, Koji Hanazawa, Koji Goto, Takahiro Doi, Toshihiro Tamura, Koh Ono, Takeshi Kimura

**Affiliations:** ^1^ Department of Cardiovascular Medicine Kyoto University Graduate School of Medicine Kyoto Japan; ^2^ Department of Cardiology Tenri Hospital Tenri Japan; ^3^ Department of Cardiovascular Medicine Kyoto Medical Center Kyoto Japan; ^4^ Department of Cardiovascular Medicine Mitsubishi Kyoto Hospital Kyoto Japan; ^5^ Department of Cardiovascular Medicine, Faculty of Medicine University of Tsukuba Tsukuba Japan; ^6^ Department of Cardiovascular Medicine Osaka Red‐Cross Hospital Osaka Japan; ^7^ Department of Cardiovascular Medicine Kobe City Medical Center General Hospital Kobe Japan; ^8^ Department of Cardiovascular Medicine Shizuoka General Hospital Shizuoka Japan; ^9^ Department of Cardiovascular Medicine Hoshigaoka Medical Center Hirakata Japan; ^10^ Department of Cardiovascular Medicine Kansai Electric Power Hospital Osaka Japan; ^11^ Department of Cardiovascular Medicine Nishinomiya Watanabe Hospital Nishinomiya Japan; ^12^ Department of Cardiovascular Medicine Fukaya Red Cross Hospital Fukaya Japan

**Keywords:** atrial fibrillation, catheter ablation, left atrial appendage flow velocity, thromboembolism

## Abstract

**Background:**

Reduced left atrial appendage flow velocity (LAAFV) on transesophageal echocardiography (TEE) is recognized as a predictor of thromboembolic events (TEs) in patients with atrial fibrillation (AF). However, its long‐term prognostic value following AF ablation remains unclear.

**Methods and Results:**

We retrospectively evaluated 1521 patients undergoing AF ablation who underwent preprocedural TEE. Patients were categorized into two groups based on LAAFV: reduced (≤ 21.4 cm/s, *n* = 99) and preserved (> 21.4 cm/s, *n* = 1422). The primary outcome was TEs. Secondary outcomes included arrhythmia recurrence and major adverse cardiovascular events (MACEs), defined as a composite of all‐cause death, stroke, major bleeding, and heart failure hospitalization, as well as individual components and cardiovascular mortality. Over a mean follow‐up of 49 ± 32 months, TEs occurred in 19 patients (1.2%). The 5‐year cumulative incidence of TEs was significantly higher in the reduced LAAFV group (6.7% vs. 0.9%, *p* < 0.0001), despite a lower rate of anticoagulation discontinuation (20.1% vs. 57.1%, *p* < 0.0001). Multivariable analysis identified reduced LAAFV as an independent predictor of TEs. It was also associated with higher risks for all secondary endpoints.

**Conclusions:**

Reduced preprocedural LAAFV is associated with adverse long‐term clinical outcomes after AF ablation, including a significantly increased risk of thromboembolic events.

## Graphical abstract

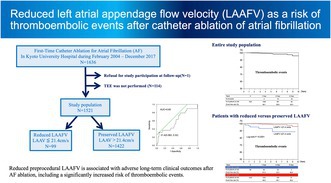

**Abbreviations**
AF: atrial fibrillation
LAA: left atrial appendage
LAAFV: left atrial appendage flow velocity
MACEs: major adverse cardiovascular events
OAC: oral anticoagulation
ROC: receiver operating characteristic
SEC: spontaneous echo contrast
TEE: transesophageal echocardiography
TEs: thromboembolic events
TIA: transient ischemic attack

## 1 Introduction

Atrial fibrillation (AF) is the most common arrhythmia causing thromboembolic events (TEs), such as ischemic stroke and systemic embolism. Oral anticoagulation (OAC) is recommended for AF patients with CHA_2_DS_2_‐VASc score of ≥ 1 in the current guidelines [1]. Also, several additional risk factors for AF‐related TEs have been reported, which include renal dysfunction, hypertrophic cardiomyopathy, persistent AF, and left atrium dilatation [1]. The left atrial appendage (LAA) is the main source of TEs in AF patients [2], and LAA flow velocity (LAAFV) measured by transesophageal echocardiography (TEE) has been recognized as a significant predictor of TEs [3, 4, 5, 6]. Catheter ablation is an established rhythm‐control therapy of AF, which maintains sinus rhythm in the majority of patients and thereby reduces the risks for TEs, death, and heart failure hospitalization [7, 8, 9]. Although several reports have shown higher rates of arrhythmia recurrence after AF ablation in patients with reduced LAAFV [10, 11], no reports have focused on the prognostic impact of preprocedural LAAFV on the comprehensive clinical outcomes including TEs after AF ablation. Consequently, we explored the clinical impact of reduced LAAFV in a large cohort of patients undergoing first‐time catheter ablation for AF.

## 2 Methods

### 2.1 Study Design

This was a retrospective, single‐center observational study. Between February 2004 and December 2017, a total of 1636 patients underwent first radiofrequency catheter ablation for AF in Kyoto University Hospital. Excluding 114 patients who did not undergo TEE before the procedure and 1 patient who refused study participation, the present study enrolled 1521 patients. Written informed consent for the radiofrequency catheter ablation procedure and follow‐up was obtained from all patients. Follow‐up information was obtained by a hospital‐chart review and/or telephone contact with the patient, relatives, and/or referring practitioners. The study protocol was approved by the institutional review board of Kyoto University Hospital.

### 2.2 Transesophageal Echocardiography

Preprocedural TEE was performed within 72 h prior to the ablation procedure. LAA images were obtained both in the basal short‐axis view with a transverse scan and in the 2‐chamber view with a vertical scan. We evaluated the presence or absence of thrombi in the left atrium as well as LAAFV and spontaneous echo contrast (SEC). The LAAFV was the peak of the emptying flow interrogated by pulsed‐wave Doppler in which the sample volume was deployed in the proximal third of the LAA. The SEC was graded from 0 to 4, and severe SEC (dense smoke) was defined as grade 3 to 4 SEC [4].

### 2.3 Ablation Protocol and Follow‐Up

The details of the radiofrequency catheter ablation procedure have been described elsewhere [12]. In brief, the procedure was performed under conscious sedation with propofol and/or dexmedetomidine. The activated clotting time was kept > 300 s with unfractionated heparin after the trans‐septal puncture. An extensive encircling pulmonary vein isolation was performed with the double circular catheter method, placing 2 circular catheters in the ipsilateral superior and inferior pulmonary veins. Continuous fractionated atrial electrocardiogram ablation and left atrial linear ablation such as mitral isthmus and left atrial roof lines were performed whenever appropriate. Tricuspid valve isthmus ablation was routinely performed regardless of the presence of previous typical atrial flutter. The superior vena cava was isolated whenever necessary.

After the ablation procedure, OAC was continued for at least 3 months. Thereafter, whether to continue OAC or not was left to the discretion of the attending physician, which was basically based on the recommendations in the consensus document [13]. Adherence to oral anticoagulation therapy was primarily assessed based on medical records of each clinical visit and/or telephone contact with the patient, relatives, and/or referring practitioners. A repeat ablation procedure was considered when recurrent atrial tachyarrhythmias were detected after the blanking period of 3 months after ablation. A 12‐lead electrocardiogram was routinely measured at each clinical visit, and 24‐h Holter monitoring was recommended at 3, 6, and 12 months and yearly thereafter.

### 2.4 Definitions and Outcome Measures

The primary outcome measure of the present study was TEs, which included ischemic stroke, transient ischemic attack (TIA), and systemic embolism. The key secondary outcome measures were recurrent atrial tachyarrhythmias and major adverse cardiovascular events (MACEs) defined as a composite of all‐cause death, stroke, major bleeding, and heart failure hospitalization. Other secondary endpoints included individual components of MACEs and cardiovascular mortality. Recurrent atrial tachyarrhythmias were defined as documented AF and/or atrial tachycardia lasting for > 30 s or those requiring repeat ablation procedures with a blanking period of 90 days after the ablation procedure. Stroke was defined as a neurological deficit requiring hospitalization with symptoms lasting for ≥ 24 h. Ischemic and hemorrhagic strokes were distinguished by imaging studies. TIA was defined as sudden focal neurologic deficits with symptoms lasting for < 24 h. Major bleeding was defined as GUSTO moderate to severe bleeding [14]. AF was classified into paroxysmal (lasting < 7 days) and non‐paroxysmal (lasting ≥ 7 days) AF. Discontinuation of OAC during follow‐up was regarded as present when it was intended to be permanent.

The LAAFV was recognized as reduced when it was equal to or below the cutoff value to predict TEs, which was determined by the receiver operating characteristic (ROC) curve analysis (Figure S1). The patients were divided into 2 groups based on the LAAFV: the reduced LAAFV group and the preserved LAAFV group.

### 2.5 Statistical Analysis

Categorical variables are presented as numbers with percentages and were compared with the chi‐square test or Fisher's exact test. Continuous variables are presented as means with standard deviations or medians with the interquartile ranges and were compared using Student's *t*‐test or the Wilcoxon rank sum test based on their distributions. We used the Kaplan–Meier method to estimate the cumulative incidence and event‐free rates, and the differences were assessed with the log‐rank test. The Cox proportional hazard model was used in the multivariable analysis to identify independent predictors of TEs. Hazard ratios with 95% confidence intervals were presented. Continuous variables were dichotomized by medians or clinically meaningful values. CHA_2_DS_2_‐VASc score and clinical parameters with *p* values of < 0.1 in the univariable analysis were incorporated into the multivariable models. Because of the limited number of TEs, we further reduced the number covariates using the forward and backward stepwise methods, with inclusion and exclusion *p* values of < 0.1 and < 0.05, respectively. Statistical analyses were performed using JMP pro 14 (SAS Institute Inc., Cary, NC) software. All analyses were two‐tailed, and a *p* value of < 0.05 was considered statistically significant.

## 3 Results

The optimal cutoff value of LAAFV to predict TEs determined by the ROC curve analysis was 21.4 cm/s with an area under curve of 0.60 (Figure S1). Thus, the study population was divided into 2 groups: 99 patients with reduced (≤ 21.4 cm/s) LAAFV and 1422 patients with preserved (> 21.4 cm/s) LAAFV.

The baseline characteristics of the study patients comparing reduced versus preserved LAAFV groups are summarized in Table 1. Mean age was 65.0 ± 9.6 years and 936 patients (61.5%) had paroxysmal AF. Patients in the reduced LAAFV group were older, and had a higher prevalence of non‐paroxysmal AF, prior heart failure, ischemic stroke and vascular disease compared to those in the preserved LAAFV group. Also, reduced LAAFV patients had higher CHADS_2_ and CHA_2_DS_2_‐VASc scores, larger left ventricular end‐diastolic diameter, lower left ventricular ejection fraction, and larger left atrium diameter.

**TABLE 1:** Baseline characteristics of the study population comparing reduced versus preserved LAAFV groups.

	Total population	Reduced LAAFV	Preserved LAAFV	*p*
	(*N* = 1521)	(*N* = 99)	(*N* = 1422)	
Age (years)	65.0 ± 9.6	67.4 ± 7.4	64.9 ± 9.7	0.01
Female (%)	432 (28.4)	36 (36.4)	396 (27.9)	0.08
Type of AF				
PAF (%)	936 (61.5)	19 (19.2)	917 (64.5)	< 0.0001
Non‐PAF (%)	585 (38.4)	80 (80.8)	505 (35.5)	
Previous heart failure (%)	170 (11.2)	32 (32.3)	138 (9.7)	< 0.0001
Previous ischemic Stroke (%)	147 (9.7)	16 (16.2)	131 (9.2)	0.04
Previous hemorrhagic stroke (%)	13 (0.9)	0 (0)	13 (0.9)	0.34
Hypertension (%)	912 (60.0)	66 (66.7)	847 (59.6)	0.16
Diabetes (%)	249 (16.4)	19 (19.2)	230 (16.2)	0.44
Vascular disease (%)	168 (11.0)	26 (26.3)	142 (10.0)	< 0.0001
Hemoglobin (g/dL)	14.0 ± 1.7	13.9 ± 1.9	14.0 ± 1.7	0.58
Creatinine	0.84 (0.71–1.00)	0.89 (0.77–1.06)	0.84 (0.70–1.00)	0.05
eGFR	66.3 ± 19.1	58.9 ± 18.3	66.7 ± 19.1	0.0005
BNP	83.5 (34.8–163.8)	193.4 (127.1–384.6)	76.8 (32.8–155.6)	< 0.0001
CHADS2 score	1.2 ± 1.1	1.7 ± 1.1	1.2 ± 1.1	< 0.0001
0	413 (27.2)	14 (14.1)	399 (28.1)	0.0007
1	615 (40.4)	38 (38.4)	577 (40.5)	
≥ 2	493 (32.4)	47 (47.5)	446 (31.4)	
CHA2DS2‐VASc score	2.1 ± 1.5	2.9 ± 1.6	2.1 ± 1.5	< 0.0001
0	203 (13.3)	6 (6.1)	197 (13.8)	0.0009
1	391 (25.7)	16 (16.1)	375 (26.4)	
≥ 2	927 (61.0)	77 (77.8)	850 (59.8)	
Transthoracic echocardiography				
LVEDD (mm)	46.5 ± 6.2	49.7 ± 8.9	46.3 ± 5.9	< 0.0001
LVEF (%)	64.2 ± 12.0	55.4 ± 18.1	64.8 ± 11.2	< 0.0001
LAD (mm)	41.6 ± 7.0	47.2 ± 6.6	41.2 ± 6.8	< 0.0001
Transesophageal echocardiography				
LAAFV (cm/s)	54.5 (37.4–72.5)	18.0 (15.8–20.0)	56.0 (41.0–73.0)	< 0.0001
Severe SEC (%)	100 (6.7)	36 (37.9)	64 (4.6)	< 0.0001
Medications at hospital discharge				
OAC	1518 (99.8)	99 (100)	1419 (99.4)	0.52
VKA	635 (41.8)	39 (39.4)	596 (42.0)	0.61
DOAC	883 (58.2)	60 (60.6)	821 (58.1)	
Antiplatelets	272 (17.9)	29 (29.3)	243 (17.1)	0.004
ACEI/ARB	635 (41.7)	47 (47.5)	581 (40.9)	0.20
Beta‐blockers	570 (37.5)	59 (59.6)	454 (31.9)	< 0.0001

Abbreviations: ACEI, angiotensin converting enzyme inhibitor; AF, atrial fibrillation; ARB, angiotensin receptor blocker; BNP, brain natriuretic peptide; DOAC, direct oral anticoagulant; LAAFV, left atrial appendage flow velocity; LAD, left atrial diameter; LVEDD, left ventricular end‐diastolic diameter; LVEF, left ventricular ejection fraction; OAC, oral anticoagulant; PAF, paroxysmal AF; SEC, spontaneous echocardiographic contrast; VKA, vitamin K antagonist.

The mean follow‐up duration was 49 ± 32 months. OAC was discontinued in 55.0% of patients at 5‐year post ablation (Figure S2‐A). The discontinuation rate of OAC was lower in the reduced LAAFV group than in the preserved LAAFV group (20.1% vs. 57.1% at 5 years, log ‐rank *p* < 0.0001) (Figure S2‐B).

**FIGURE 1:**
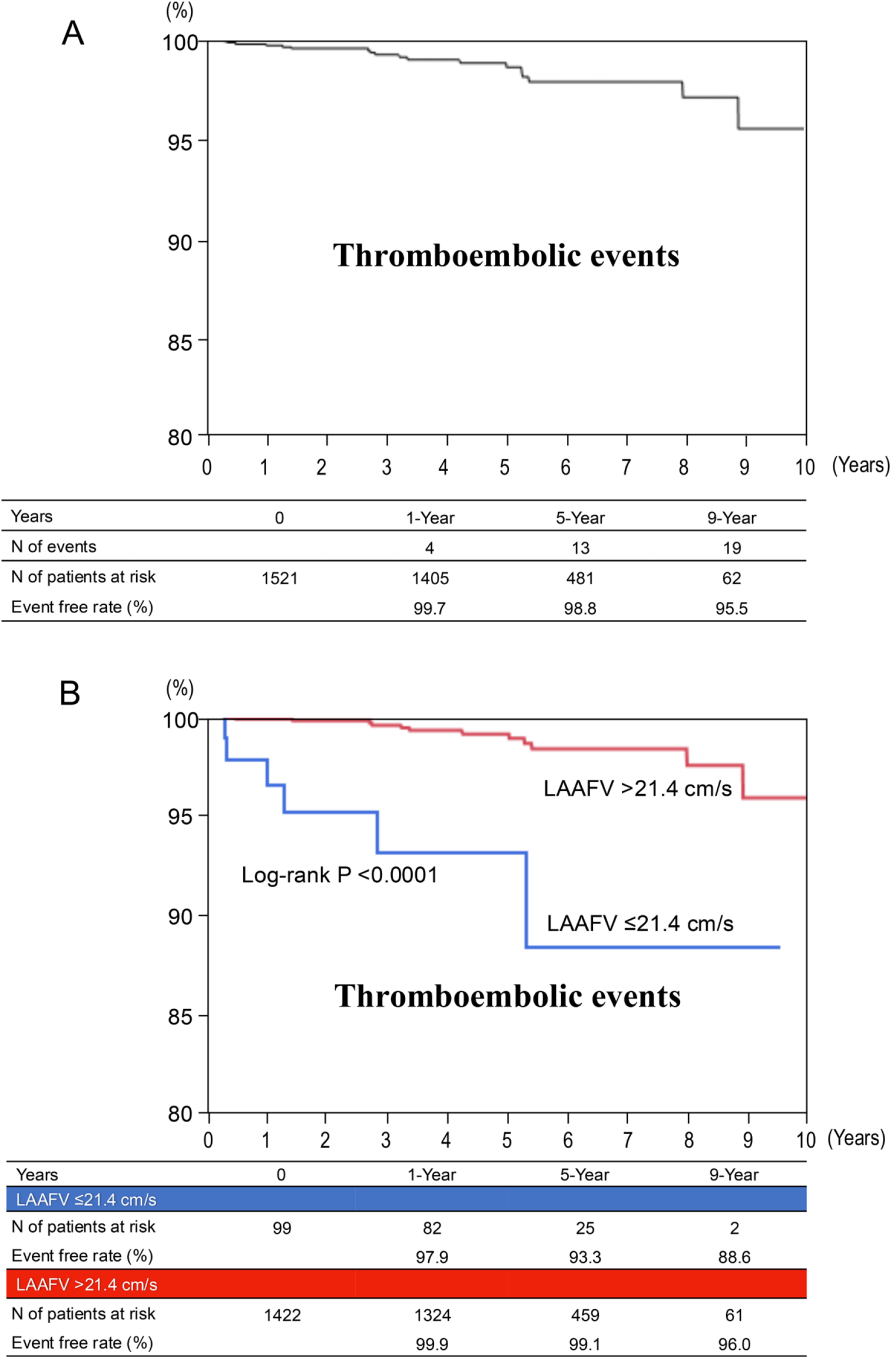
The event‐free rates from the primary endpoint of TEs: (A) in the entire study population and (B) in patients with reduced versus preserved LAAFV. The vertical axis is truncated (80%–100%) to highlight intergroup differences. LAAFV, left atrial appendage flow velocity, TEs, thromboembolic events.

The primary and secondary outcomes are summarized in Table 2. The primary outcome measure of TEs occurred in 19 patients (1.2%): 15 ischemic strokes, 3 TIAs and 1 systemic embolism. There were no patients with ≥ 2 TEs. The 5‐year cumulative incidence of TEs in the entire study population was 1.2% (Figure 1A). Further details of the TEs are shown in the Table 3. There was no acute event within 1 month after the procedure, and the median time from ablation to TE was 47.7 (3.6–106.8) months. OAC had been discontinued at the time of the TEs in 7 patients (36.8%) who had had no documented recurrent atrial tachyarrhythmias. Also, OAC had been interrupted in 3 patients (15.8%) because of gastrointestinal bleeding in 2 patients and elective surgery in 1 patient. The remaining 9 patients (47.4%) was on OAC at the time of the event, although the intensity of OAC was suboptimal in 3 patients.

**TABLE 2:** Primary and secondary outcomes.

	Total population	Reduced LAAFV	Preserved LAAFV	Log‐rank *p*
	(*N* = 1521)	(*N* = 99)	(*N* = 1422)	
Thromboembolic events (TEs)	19 (1.2%)	6 (6.7%)	13 (0.9%)	< 0.0001
Ischemic stroke	15 (1.0%)	4 (3.7%)	11 (0.8%)	< 0.0001
TIA	3 (0.2%)	1 (1.4%)	2 (0.1%)	0.02
Systemic embolism	1 (0.1%)	1 (2.1%)	0 (0%)	< 0.0001
Recurrent atrial tachyarrhythmias	575 (43.1%)	53 (64.3%)	522 (41.6%)	< 0.0001
MACEs^a^	111 (9.1%)	16 (20.8%)	95 (8.3%)	< 0.0001
All‐cause death	59 (5.3%)	8 (9.4%)	51 (5.1%)	0.003
Stroke	21 (1.2%)	5 (5.0%)	16 (0.9%)	0.0001
Major bleeding	21 (1.7%)	5 (8.0%)	16 (1.3%)	0.0002
Heart failure hospitalization	40 (3.3%)	11 (17.0)	29 (2.6%)	< 0.0001
Cardiovascular death	21 (1.8%)	5 (5.7%)	16 (1.5%)	0.0001

*Note:* Numbers of events during follow‐up and 5‐year cumulative incidence rates comparing the reduced versus preserved LAAFV groups are presented.

Abbreviations: LAAFV, left atrial appendage flow velocity; MACEs, major adverse cardiovascular events; TIA, transient ischemic attack.

^a^ Defined as a composite of all‐cause death, stroke, major bleeding, and heart failure hospitalization.

**TABLE 3:** Details of TEs.

No.	Type of TE	LAAFV (cm/s)	LAAFV group	Age	Sex	CHA_2_DS_2_‐VASc score	RAT before TE	AF type	Time from ABL to TE (Months)	OAC at TE	PT‐INR/APTT at TE
1	Ischemic stroke	15	Reduced	60	M	1	Yes	Non‐PAF	12	Interrupted	—
2	Ischemic stroke	18	Reduced	65	M	4	No	PAF	63	Discontinued	—
3	Systemic embolism	19	Reduced	65	F	1	Yes	Non‐PAF	33	VKA	1.80/41.6
4	Ischemic stroke	19	Reduced	69	F	4	Yes	Non‐PAF	15	Rivaroxaban	NA
5	Ischemic stroke	21	Reduced	71	F	4	Yes	Non‐PAF	3	Interrupted	—
6	TIA	21	Reduced	74	M	3	Yes	Non‐PAF	3	Interrupted	—
7	Ischemic stroke	29	Preserved	70	F	3	No	PAF	63	Discontinued	—
8	TIA	44	Preserved	62	F	1	No	PAF	95	Discontinued	—
9	Ischemic stroke	49	Preserved	74	M	2	Yes	PAF	106	VKA	1.48/39.6
10	Ischemic stroke	50	Preserved	64	M	3	No	PAF	51	Discontinued	—
11	Ischemic stroke	52	Preserved	60	M	3	Yes	PAF	33	VKA	NA
12	TIA	52	Preserved	70	M	2	Yes	PAF	17	VKA	2.10/41.4
13	Ischemic stroke	56	Preserved	76	F	6	No	PAF	5	VKA	NA
14	Ischemic stroke	56	Preserved	62	F	2	Yes	Non‐PAF	32	VKA	1.20/30.1
15	Ischemic stroke	59	Preserved	74	F	2	Yes	PAF	60	Dabigatran	1.35/47.4
16	Ischemic stroke	68	Preserved	72	M	4	No	PAF	3	VKA	2.07/40.1
17	Ischemic stroke	71	Preserved	70	M	2	No	PAF	38	Discontinued	—
18	Ischemic stroke	111	Preserved	59	M	0	No	PAF	40	Discontinued	—
19	Ischemic stroke	122	Preserved	65	M	0	No	PAF	64	Discontinued	—

Abbreviations: ABL, ablation; AF, atrial fibrillation; APTT, activated partial thromboplastin time; LAAFV, left atrial appendage flow velocity; NA, not available; OAC, oral anticoagulant; PAF, paroxysmal AF; PT‐INR, prothrombin time‐international normalized ratio; RAT, recurrent atrial tachyarrhythmias; TE, thromboembolic event; TIA, transient ischemic attack; VKA, vitamin K antagonist.

**FIGURE 2:**
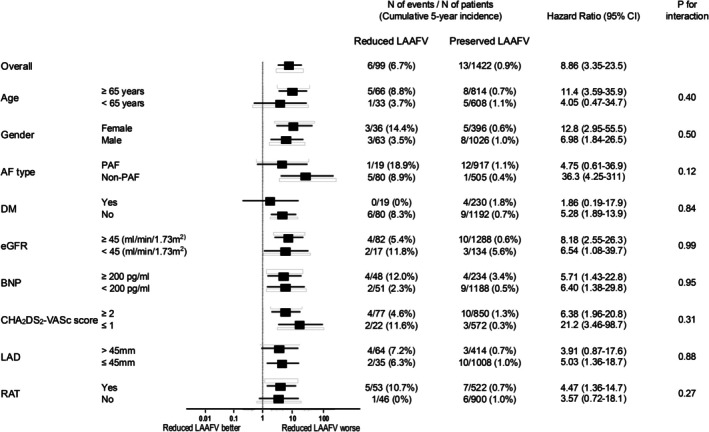
The risks of TEs comparing reduced versus preserved LAAFV in patient subgroups. LAAFV, left atrial appendage flow velocity; TEs, thromboembolic events.

The cumulative incidence rate of the primary endpoint of TEs was significantly higher in the reduced LAAFV group than in the preserved LAAFV group (6.7% vs. 0.9% at 5 years, log‐rank *p* < 0.0001) (Table 2 and Figure 1B), despite the lower rate of discontinuation of OAC in the reduced LAAFV group. The higher incidence of TEs in the reduced LAAFV group was consistent through the subgroups of patients (Figure 2), including those divided by CHA_2_DS_2_‐VASc score, recurrent atrial tachyarrhythmias, and type of AF. The baseline characteristics comparing patients with and without TEs are shown in Table S1.

The predictors of the primary endpoint of TEs in the univariable analysis were listed in the Table S2. In addition to the reduced LAAFV, severe SEC in the preprocedural TEE was also strongly associated with TEs. However, the area under curve in the ROC curve analysis of SEC to predict TEs was only 0.50. Also, the chi‐square of severe SEC in the univariable Cox analysis for TEs was smaller than that of reduced LAAFV. Furthermore, there were 64 patients with missing data of SEC. Given the substantial correlation between the LAAFV and SEC (*r* = 0.49. *p* < 0.0001) as well as larger predictive value of LAAFV for TEs compared to SEC, only reduced LAAFV was incorporated into the multivariable model as a parameter representing reduced function of left atrium and LAA assessed by preprocedural TEE. Other covariables included renal dysfunction, high level of serum brain natriuretic peptide, usage of beta‐blockers and warfarin, anemia, and CHA_2_DS_2_‐VASc score of ≥ 2. As a result, in the multivariable analysis, the reduced LAAFV was an independent predictor of TEs, along with renal dysfunction (Table 4).

**TABLE 4:** Predictors of TEs after AF ablation.

Variable	Univariable analysis		Multivariable analyses					
			Model‐1		Model‐2		Final model	
	Unadjusted HR (95% CI)	*p*	Adjusted HR (95% CI)	*p*	Adjusted HR (95% CI)	*p*	Adjusted HR (95% CI)	*p*
LAAFV ≤ 21.4 cm/s	8.86 (3.35–23.5)	< 0.0001	6.03 (2.11–17.2)	0.0008	8.13 (3.05–21.7)	< 0.0001	7.86 (2.93–21.0)	< 0.0001
eGFR < 45 mL/min/1.73m^2^	6.06 (2.11–17.4)	0.0008	3.58 (1.04–12.3)	0.043	5.44 (1.86–15.9)	0.002	4.88 (1.62–14.8)	0.005
BNP ≥ 200 pg/mL	4.50 (1.79–11.3)	0.001	1.75 (0.59–5.14)	0.31				
Use of beta‐blockers	3.20 (1.25–8.15)	0.015	1.97 (0.74–5.26)	0.10				
Use of VKA	3.28 (0.90–12.0)	0.048	2.97 (0.80–11.0)	0.10				
Hb < 11 g/dL	3.84 (0.88–16.8)	0.07	1.16 (0.22–6.13)	0.86				
CHA_2_DS_2_‐VASc score ≥ 2	2.11 (0.75–5.90)	0.15	1.37 (0.46–4.08)	0.57			1.45 (0.49–4.26)	0.50

*Note:* Parameters with a *p* value of < 0.1 in the univariable analysis and CHA_2_DS_2_‐VASc score were incorporated into the multivariate models. In the Model‐1, all parameters were incorporated. In the Model‐2, the forward and backward stepwise methods were developed, leading to same results. In the Final Model, CHA_2_DS_2_‐VASc score was added to the Model‐2. Other abbreviations as in Table 1.

Abbreviations: CI, confidence interval; Hb, hemoglobin; HR, hazard ratio; TEs, thromboembolic events.

**FIGURE 3:**
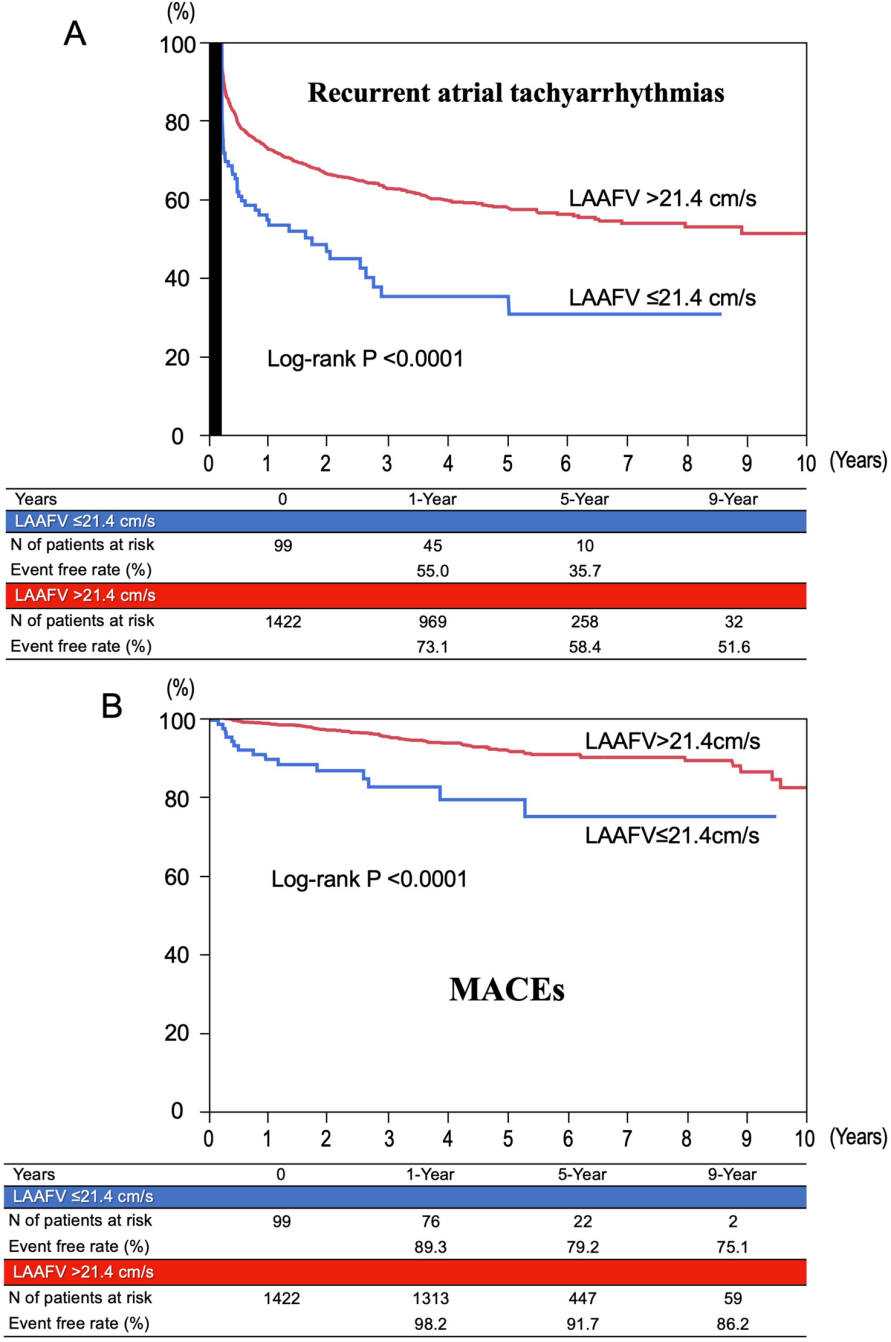
The event‐free rates from (A) recurrent atrial tachyarrhythmias and (B) MACEs, comparing patients with reduced versus preserved LAAFV. LAAFV, left atrial appendage flow velocity; MACEs, major adverse cardiovascular even.

The key secondary endpoints, recurrent atrial tachyarrhythmias, and MACEs, were also more frequent in the reduced LAAFV group than in the preserved LAAFV group (63.8% vs. 41.3% at 5 years, log ‐rank *p* < 0.0001, and 20.7% vs. 8.4% at 5 years, log ‐rank *p* < 0.0001) (Table 2 and Figure 3). Reduced LAAFV was also associated with higher incidence rates of all other secondary endpoints, including all‐cause and cardiovascular deaths, stroke, major bleeding, and heart failure hospitalization, as compared to preserved LAAFV (Table 2 and Figure S3).

## 4 Discussion

The main findings of the present study were the following: (1) the incidence of TEs after catheter ablation of AF was significantly higher in the reduced LAAFV group than in the preserved LAAFV group, despite the much lower rate of discontinuation of OAC in the reduced LAAFV group. (2) the excess risk of TEs in the reduced LAAFV group relative to the preserved LAAFV group was consistent across the subgroups of patients including those divided by CHA_2_DS_2_‐VASc score, arrhythmia recurrence, and AF type, (3) the reduced LAAFV was an independent predictor of TEs in the multivariable analysis, (4) the reduced LAAFV was also associated with higher risks for recurrent atrial tachyarrhythmias and MACEs, (5) the cumulative risks for all‐cause and cardiovascular deaths, stroke, major bleeding, and heart failure hospitalization were also significantly higher in the reduced LAAFV group.

Although reduced LAAFV is recognized as a significant predictor of TEs in AF patients, [3, 4, 5, 6] there have been no reports evaluating the association between preprocedural LAAFV and TEs after catheter ablation of AF. Only 1 report by Gedikli et al. showed that severe SEC (dense smoke) in preprocedural TEE was associated with late TEs after AF ablation regardless of CHA_2_DS_2_‐VASc score [15], which was concordant with the present study. Although both reduced LAAFV and severe SEC reflect impaired left atrium and LAA function, LAAFV has advantages over SEC in terms of quantitativity and objectivity. Actually, in the present study, both reduced LAAFV and severe SEC were significantly associated with TEs in the univariable analysis, but the predictive value of LAAFV for TEs was larger than that of SEC. The cutoff values of LAAFV in previous studies assessing the relation between LAAFV and TEs or LAA thrombus in AF patients ranged from 20 to 30 cm/s [3, 4, 5, 6]. In the present study, we set the cutoff value of LAAFV as 21.4 cm/s based on the results of the ROC curve analysis, so that the analyses would not be arbitrary. We also performed sensitivity analyses setting the cutoff values of LAAFV as 20, 25 and 30 cm/s, and the results were generally consistent with the original analysis (Table S3).

The discontinuation rate of OAC in the present study was 55.0% at 5 years post ablation (Figure S2‐A). Although whether to discontinue OAC or not during follow‐up was left to the discretion of the attending physician, the decision was likely to be based mainly on the CHA_2_DS_2_‐VASc score, but also on the presence or absence of arrhythmia recurrence and AF type (Figure S2‐C–G). Presumably because of the higher CHA_2_DS_2_‐VASc score as well as the higher arrhythmia recurrence rate and higher prevalence of non‐paroxysmal AF, the discontinuation rate of OAC was far lower in the reduced LAAFV patients than in the preserved LAAFV patients (20.1% vs. 57.1% at 5 years, log ‐rank *p* < 0.0001) (Figure S2‐B). Because reduced LAAFV was an independent predictor of TEs regardless of CHA_2_DS_2_‐VASc score, arrhythmia recurrence, and type of AF (Figure 2 and Table 4), long‐term continuation of OAC should be considered in patients with reduced LAAFV, even if they have a low CHA_2_DS_2_‐VASc score or maintained sinus rhythm after AF ablation. Kusa et al. reported that the majority of patients with reduced (≤ 40 cm/s) LAAFV before AF ablation had improved but still reduced LAAFV beyond 6 months after ablation, despite successfully maintained sinus rhythm [16]. Actually, in the present study, 13 out of 19 patients (68.4%) with TEs had had no documented arrhythmia recurrences at the time of the event.

Reduced LAAFV has been reported to be associated with higher arrhythmia recurrence after AF ablation [10, 11]. In line with the previous studies, our study also showed that reduced LAAFV was strongly associated with recurrent atrial tachyarrhythmias. Furthermore, reduced LAAFV was associated with higher risks for MACEs, cardiovascular mortality, and individual components of MACEs (Table 2, Figure 3, and Figure S3). To the best of our knowledge, the present study for the first time showed the negative impact of reduced preprocedural LAAFV on the comprehensive clinical outcomes including TEs after AF ablation during long‐term follow‐up.

The present study has several limitations. First, this was a single‐center observational study with inherent biases. Second, the TEs could have been partly not AF‐related thromboembolism but due to artery atherosclerosis. Third, during long‐term follow‐up beyond the first year, annual 24‐h Holter monitoring might have been inadequate to detect arrhythmia recurrence. Finally, due to the limited number of TEs, the number of covariates in the multivariable model was reduced to avoid overfitting, which may have led to inadequate adjustment of baseline confounders.

## 5 Conclusions

The reduced preprocedural LAAFV was independently associated with TEs after AF ablation. The decision to discontinue OAC or not after AF ablation should be based not only on the CHADS_2_ or CHA_2_DS_2_‐VASc scores, bleeding risk, and patients' preference or adherence but also on the preprocedural LAAFV.

## Funding

The authors have nothing to report.

## Disclosure

Dr. Koh Ono is a member of Circulation Journal's editorial team.

## Ethics Statement

The present study was approved by Kyoto University Graduate School and Faculty of Medicine, Ethics Committee. Reference number: E2492.

## Conflicts of Interest

The authors declare no conflicts of interest.

## Supporting information


**Table S1:** Baseline characteristics of patients with and without TEs.
**Table S2:** Predictors of TEs after AF ablation in the univariable analysis.
**Table S3:** Sensitivity analysis setting the cutoff values of LAAFV as 20, 25 and 30 cm/s using the final multivariable model in Table 4.
**Figure 1**. The ROC curve analysis to determine the optimal cutoff value of LAAFV to predict TEs.
The value 21.4 represents the optimal cutoff of LAAFV, while the values in parentheses (0.062, 0.32) indicate the 95% confidence interval for the AUC.
**Figure S2:** Cumulative incidence rates of OAC discontinuation.
**Figure S3:** Event‐free survival curves from the individual components of MACEs and cardiovascular death comparing patients with reduced versus preserved LAAFV.
