# Supplementary material for: Reduced Left Atrial Appendage Flow Velocity as a Risk of Thromboembolic Events After Catheter Ablation of Atrial Fibrillation
Source: J Arrhythm. 2025 Dec 10;41(6):e70233. doi: 10.1002/joa3.70233 (PMC12695476; doi:10.1002/joa3.70233)
Supplement: Supplementary file 1 — Table S1: Baseline characteristics of patients with and without TEs. Table S2: Predictors of TEs after AF ablation in the univariable analysis. Table S3: Sensitivity analysis setting the cutoff values of LAAFV as 20, 25 and 30 cm/s using the final multivariable model in Table 4. Figure 1. The ROC curve analysis to determine the optimal cutoff value of LAAFV to predict TEs. The value 21.4 represents the optimal cutoff of LAAFV, while the values in parentheses (0.062, 0.32) indicate the 95% confidence interval for the AUC. Figure S2: Cumulative incidence rates of OAC discontinuation. Figure S3: Event‐free survival curves from the individual components of MACEs and cardiovascular death comparing patients with reduced versus preserved LAAFV. [file JOA3-41-e70233-s001.docx]

**Supplementary Materials**

**Supplementary Tables**

**Supplementary Table 1 ---------------------------------------------------- Page 2**

**Supplementary Table 2 ---------------------------------------------------- Page 4**

**Supplementary Table 3 ---------------------------------------------------- Page 5**

**Supplementary Figures**

**Supplementary Figure 1 -------------------------------------------------- Page 6**

**Supplementary Figure 2 -------------------------------------------------- Page 7**

**Supplementary Figure 3 -------------------------------------------------- Page 8**

**Supplementary Table 1.**

**Baseline characteristics of patients with and without TEs.**

|  | TEs | No TEs | P value |
| --- | --- | --- | --- |
|  | (N=19) | (N=1502) |  |
| Age (years) | 67.4±5.4 | 65.0±9.7 | 0.26 |
| Female (%) | 8 (42.1) | 424 (28.2) | 0.20 |
| Type of AF |  |  | 0.53 |
| PAF (%) | 13 (68.4) | 923 (61.4) |  |
| Non-PAF (%) | 6 (31.6) | 579 (38.6) |  |
| Previous heart failure (%) | 3 (15.8) | 167 (11.1) | 0.52 |
| Previous ischemic stroke (%) | 3 (15.8) | 144 (9.6) | 0.36 |
| Previous hemorrhagic stroke (%) | 0 (0) | 13 (0.87) | 0.68 |
| Hypertension (%) | 11 (57.9) | 902 (60.1) | 0.85 |
| Diabetes (%) | 4 (21.1) | 245 (16.3) | 0.58 |
| Vascular disease (%) | 4 (21.1) | 164 (10.9) | 0.16 |
| Hemoglobin | 12.9±1.6 | 14.0±1.7 | 0.004 |
| eGFR | 54.0±26.8 | 66.5±19.0 | 0.005 |
| BNP | 165.0 (49.8-463) | 83.3 (34.6-162.1) | 0.02 |
| CHADS2 score |  |  | 0.91 |
| 0 | 5 (26.3) | 408 (27.1) |  |
| 1 | 7 (36.8) | 608 (40.5) |  |
| ≥2 | 7 (36.8) | 486 (32.4) |  |
| CHA2DS2-VASc score |  |  | 0.51 |
| 0 | 2 (10.5) | 201 (13.4) |  |
| 1 | 3 (15.8) | 388 (25.8) |  |
| ≥2 | 14 (73.7) | 913 (60.8) |  |
| Transthoracic echocardiography |  |  |  |
| LVEDD (mm) | 47.5±9.8 | 46.5±6.1 | 0.49 |
| LVEF (%) | 62.8±15.4 | 64.2±12.0 | 0.62 |
| LAD (mm) | 42.7±6.3 | 41.6±7.0 | 0.50 |
| Transesophageal echocardiography |  |  |  |
| LAAFV (cm/s) | 50.0 (21.0-59.0) | 54.0 (37.7-72.0) | 0.13 |
| LAAFV ≤21.4 cm/s (%) | 6 (31.6) | 93 (6.2) | 0.0009 |
| Severe SEC (%) | 4 (22.2) | 96 (6.5) | 0.03 |
| Medications at hospital discharge |  |  |  |
| OAC (%) | 19 (100) | 1499 (99.8%) | 0.86 |
| VKA (%) | 16 (84.2) | 619 (41.3) | 0.0002 |
| DOAC (%) | 3 (15.8) | 880 (58.7) |  |
| Antiplatelets (%) | 7 (36.8) | 265 (17.6) | 0.049 |
| ACEI / ARB (%) | 5 (26.3) | 623 (41.5) | 0.18 |
| Beta-blockers (%) | 12 (63.2) | 501 (33.4) | 0.009 |

ACEI=angiotensin converting enzyme inhibitor, AF=atrial fibrillation, ARB=angiotensin receptor blocker, DOAC=direct oral anticoagulant, LAAFV=left atrial appendage flow velocity, LAD=left atrial diameter, LVEDD=left ventricular end-diastolic diameter, LVEF=left ventricular ejection fraction, OAC=oral anticoagulation, PAF=paroxysmal AF, SEC=spontaneous echocardiographic contrast, TE=thromboembolic event, VKA=vitamin K antagonist.

**Supplementary Table 2. Predictors of TEs after AF ablation in the univariable analysis**

| Variable | Unadjusted HR | 95% CI | Chi-square | P value |
| --- | --- | --- | --- | --- |
| Age ≥65 years | 2.05 | 0.78-5.43 | 2.10 | 0.15 |
| Female | 1.74 | 0.70-4.34 | 1.43 | 0.23 |
| Non-paroxysmal AF | 1.03 | 0.39-2.74 | 0.004 | 0.95 |
| Previous heart failure | 2.25 | 0.65-7.82 | 1.62 | 0.20 |
| Previous ischemic stroke | 1.48 | 0.43-5.08 | 0.38 | 0.54 |
| Hypertension | 0.94 | 0.38-2.35 | 0.01 | 0.90 |
| Diabetes | 1.51 | 0.50-4.56 | 0.54 | 0.46 |
| Vascular disease | 2.28 | 0.76-6.88 | 2.14 | 0.14 |
| Hb < 11 g/dl | 3.84 | 0.88-16.8 | 3.19 | 0.07 |
| eGFR < 45 ml/min/1.73m^2^ | 6.06 | 2.11-17.4 | 11.24 | 0.0008 |
| BNP ≥200 pg/ml | 4.50 | 1.79-11.3 | 10.25 | 0.001 |
| CHADS2 score ≥2 | 1.41 | 0.55-3.59 | 0.50 | 0.48 |
| CHA2DS2-VASc score ≥2 | 2.12 | 0.76-5.90 | 2.05 | 0.15 |
| LVEDD > 55 mm | 2.57 | 0.75-8.86 | 2.25 | 0.13 |
| LVEF < 50 % | 1.14 | 0.26-4.96 | 0.03 | 0.86 |
| LAD > 45 mm | 1.48 | 0.58-3.76 | 0.67 | 0.41 |
| LAAFV ≤21.4 cm/s | 8.86 | 3.35-23.5 | 13.61 | <0.0001 |
| Severe SEC | 6.91 | 2.20-21.7 | 10.94 | 0.0009 |
| Use of VKA | 3.28 | 0.90-12.0 | 3.25 | 0.048 |
| Use of Antiplatelets | 2.03 | 0.79-5.18 | 2.17 | 0.14 |
| Use of ACEI/ARB | 0.51 | 0.18-1.40 | 1.71 | 0.19 |
| Use of beta-blockers | 3.20 | 1.25-8.15 | 5.97 | 0.015 |
| OAC discontinuation | 0.65 | 0.27-1.64 | 0.81 | 0.37 |
| Arrhythmia recurrence | 1.38 | 0.56-3.41 | 0.49 | 0.48 |

HR=hazard ratio, CI=confidence interval, TEs=thromboembolic events. Other abbreviations as in Supplementary Table 1.

**Supplementary Table 3.**

Sensitivity analysis setting the cutoff values of LAAFV as 20, 25 and 30 cm/s using the final multivariable model in Table 4.

| **Multivariable Analysis** | | | | | | |
| --- | --- | --- | --- | --- | --- | --- |
| Variable | Adjusted HR  (95% CI) | p | Adjusted HR  (95% CI) | p | Adjusted HR  (95% CI) | p |
| LAAFV  20 cm/s    25 cm/s  30 cm/s | 5.20 (1.70-15.9) | 0.004 | 4.85 (1.82-12.9) | 0.002 | 3.34 (1.30-8.57) | 0.01 |
| eGFR < 45 ml/min/1.73 m^2^ | 5.11 (1.71-15.3) | 0.004 | 5.04 (1.67-15.2) | 0.004 | 4.66 (1.55-14.1) | 0.006 |
| CHA2DS2-VASc score ≥ 2 | 1.47 (0.50-4.33) | 0.48 | 1.56 (0.53-4.57) | 0.42 | 1.60 (0.54-4.72) | 0.39 |

All abbreviations as in Supplementary Table 1 and Supplementary Table 2.

**Supplementary Figure 1.**

The ROC curve analysis to determine the optimal cutoff value of LAAFV to predict TEs.
The value **21.4** represents the optimal cutoff of LAAFV, while the values in parentheses **(0.062, 0.32)** indicate the**95% confidence interval**for the**AUC**.


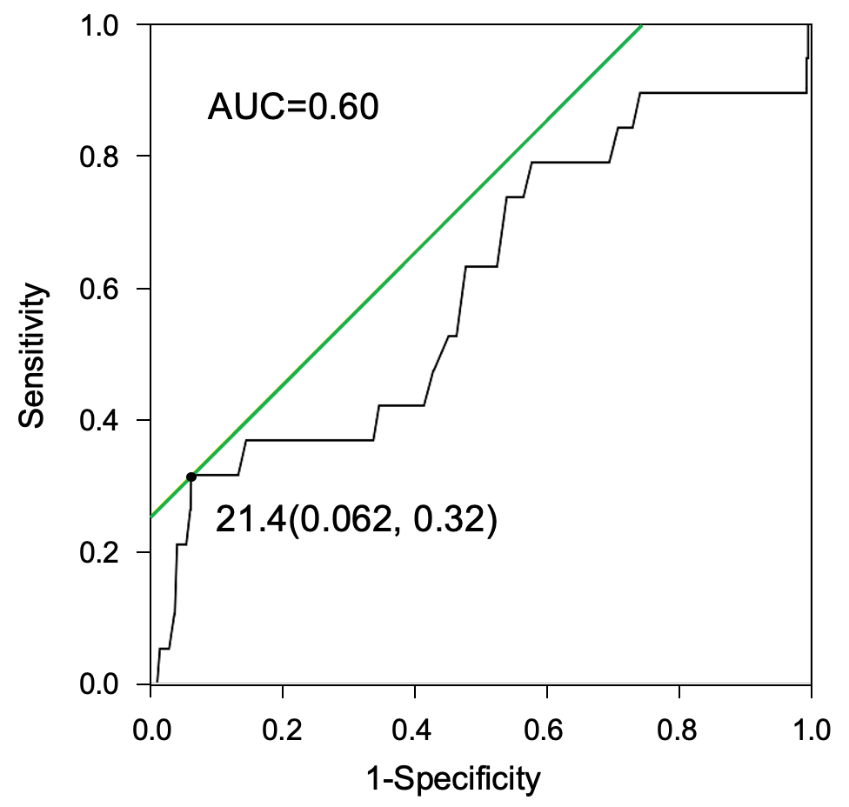


AUC=area under curve, LAAFV=left atrial appendage flow velocity, ROC=receiver operating characteristic, TEs=thromboembolic events.

**Supplementary Figure 2. Cumulative incidence rates of OAC discontinuation**

Cumulative incidence rates of discontinuation of OAC: (A) in the entire study population, (B) comparing patients with reduced (≤21.4 cm/s) versus preserved (>21.4 cm/s) LAAFV, (C) comparing patients with CHA_2_DS_2_-VASc score of ≥2 versus those with CHA_2_DS_2_-VASc score of ≤1, (D) comparing 4 groups of patients divided by CHA_2_DS_2_-VASc score, (E) comparing patients with and without recurrent atrial tachyarrhythmias, and (F) comparing patients with paroxysmal AF versus non-paroxysmal AF.

1. (B)


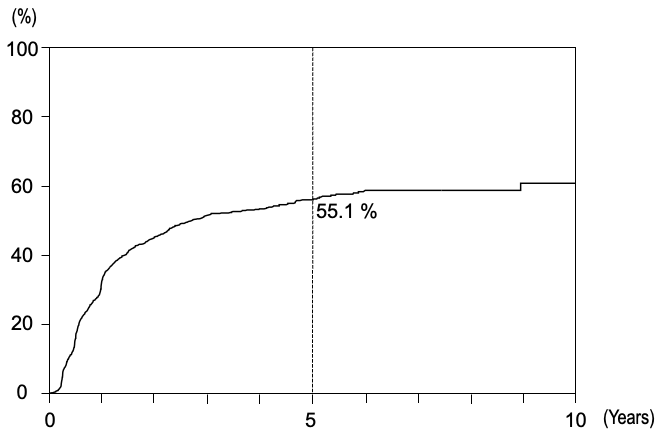

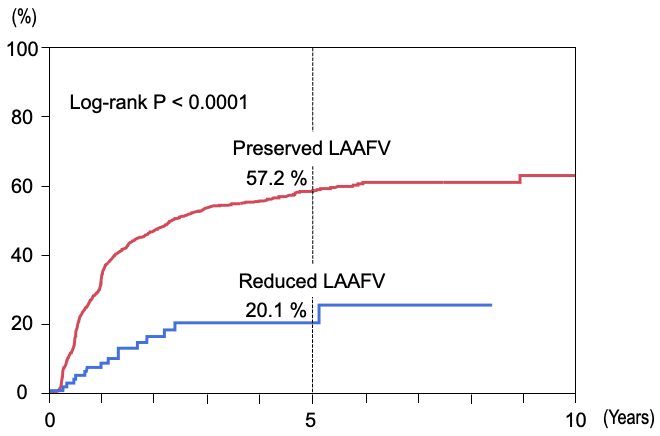


(C) (D)


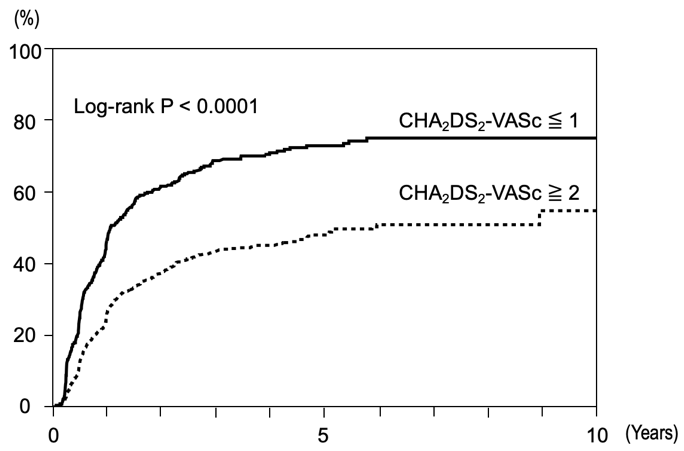

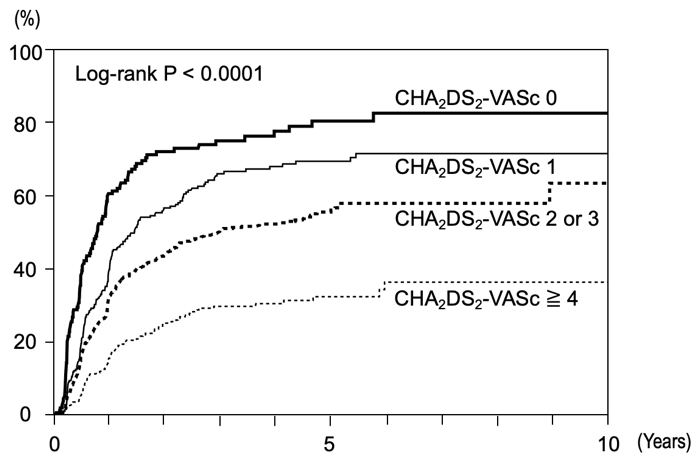


(E) (F)


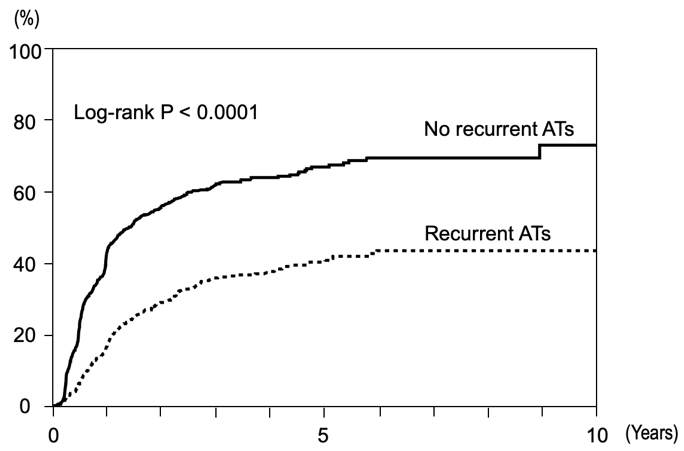

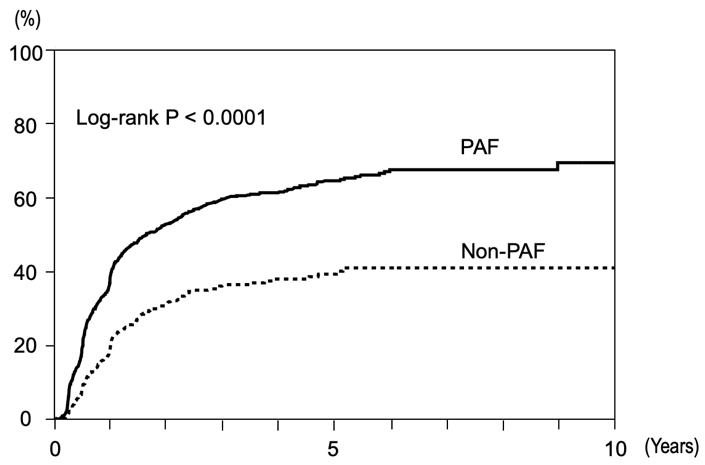


AF=atrial fibrillation, ATs=atrial tachyarrhythmias, LAAFV= left atrial appendage flow velocity, OAC= oral anticoagulation, PAF=paroxysmal AF.

**Supplementary Figure 3.**

**Event-free survival curves from the individual components of MACEs and cardiovascular death comparing patients with reduced versus preserved LAAFV.**

**All-cause-death Cardiovascular death**

**
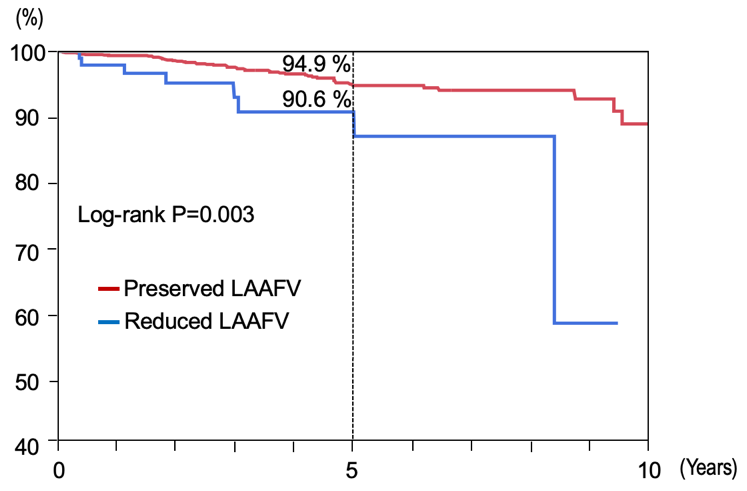

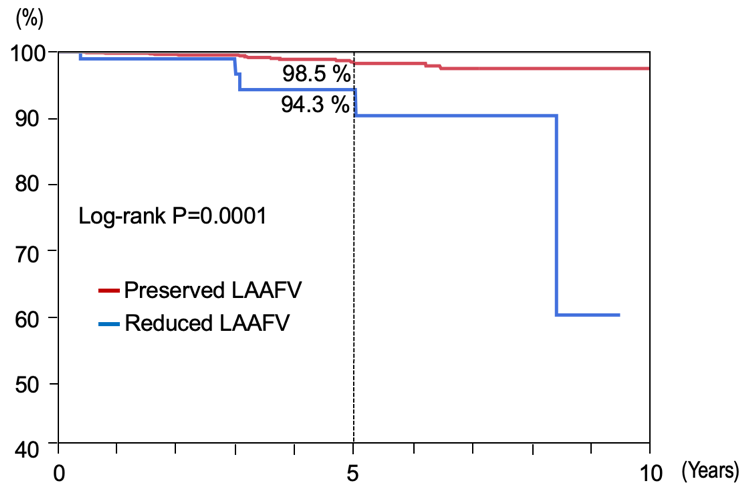
**

**Stroke Major bleeding**


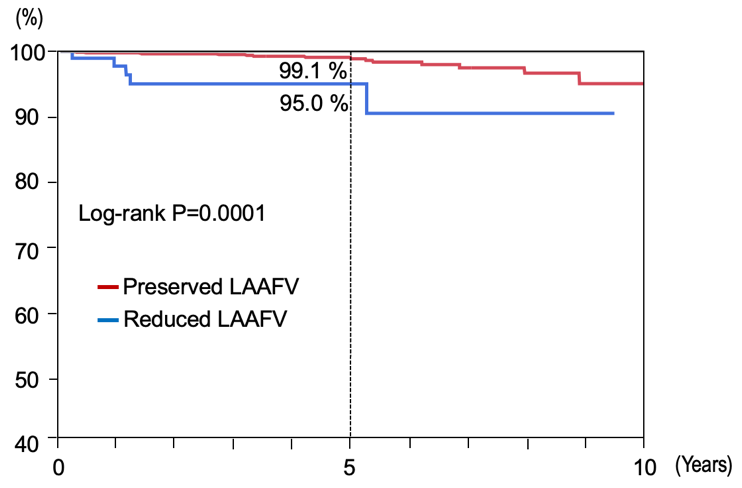

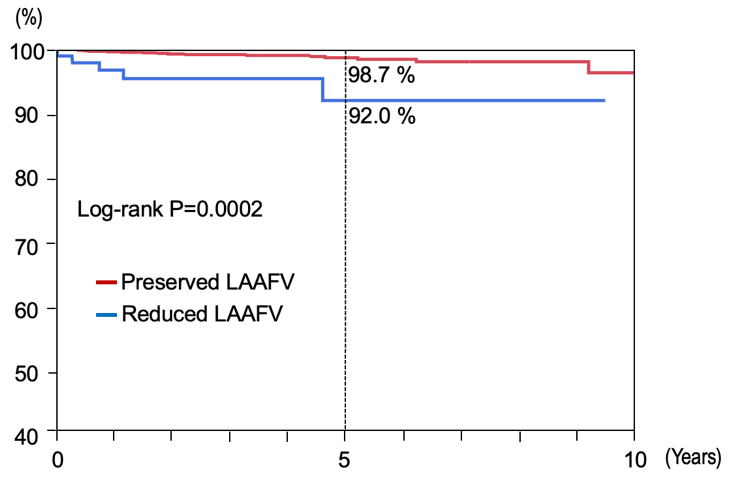


**Heart failure hospitalization**


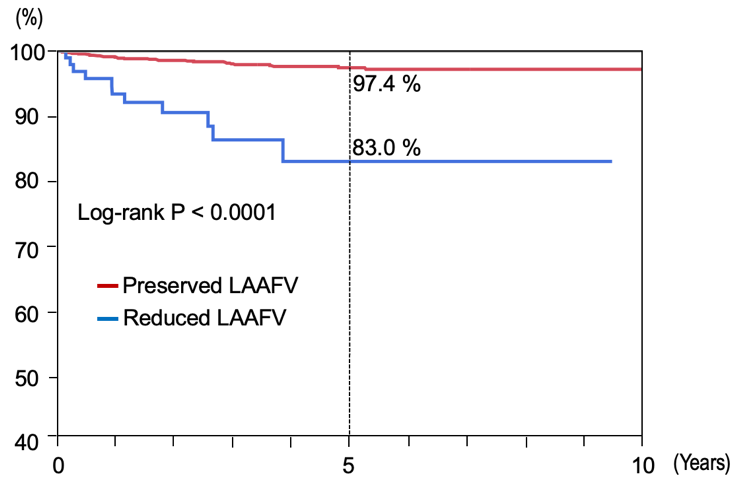


LAAFV= left atrial appendage flow velocity, MACEs= major adverse cardiovascular events.
